# Substrate Optimization in Baby Hamster Kidney Cell Culture for Foot and Mouth Disease Virus Vaccine Using the Taguchi Method

**DOI:** 10.25122/jml-2019-0040

**Published:** 2020

**Authors:** Mahmoud Hasani, Reza Golhosseini, Sayed Mahmoud Azimi, Mahdi Ardjmand, Homayon Mahravani, Shahriar Salemi Parizi

**Affiliations:** 1. Department of Chemical Engineering, Faculty of Engineering, University of Kashan, Kashan, Iran; 2. Foot and Mouth Disease Reference Laboratory, Razi Vaccine & Serum Research Institute, Agricultural Research Education and Extension Organization (AREEO), Karaj, Iran; 3. Department of Chemical Engineering, Tehran South Branch, Islamic Azad University, Tehran, Iran; 4. Department of Chemical Engineering, Faculty of Engineering, Science and Research Branch, Islamic Azad University, Tehran, Iran

**Keywords:** Baby hamster kidney, suspension, supplements, experimental design, Taguchi method, glutamine, yeast extract

## Abstract

Cell culture is one of the most commonly used techniques in the production of biological products. Many physical and chemical parameters may affect cell growth and proliferation. This study was conducted to investigate the effect of chemical components as supplements using the experimental design method, which aimed at reducing the number of experiments. For this purpose, supplements including chemical components using four levels, with three replications in suspension and batch culture conditions, were examined for 72 hours using the Taguchi experimental design method. From the experiments, it was concluded that the culture media composition had a significant impact on final cell count and pH. High concentrations of different media composition alone were insufficient to ensure higher cell count. According to the results, this insufficiency was associated with an increase of 20% in the number of final cells. In the majority of cultures, the number of final cells at 48 hours increased relative to the number of final cells at 24 hours after culturing the cells.

## Introduction

Animal cells are widely used in cell biology, cancer research, diagnostics and pharmaceuticals. Cell culture is historically used for the isolation and propagation of viruses and is categorized into two groups. One is primary cell culture, and another one is cell lines. Primary cells offer a method of choice for the isolation of viruses from the clinical specimen while cell lines are routinely used for achieving high biological titer of animal viruses [1,2].

Culturing cells and tissue is as a synthetic process for the growth, propagation and maintenance of cells for research and industrial purposes on the behavior of various animal cells which was invented in the early twentieth century and is applicable in biochemical, cytogenetic and cellular laboratories for research and diagnosis [3,4,5].

This method lacks any systematic changes due to the related stresses of animal experiments or the natural homogeneous nature of the living organism, which results in a higher quality of experiments [3]. Cell culture is a general and fundamental technique that involves separating the cell from an organ or animal tissue, and then growing it in a culture medium under synthetic conditions, rather than the cells growing within the living creature [6,7].

Baby hamster kidney (BHK) cells are most commonly used in the production of animal products, the most important of which is the production of a vaccine against Foot-and-Mouth Disease and rabies [8]. Also, BHK was used in the production of recombinant proteins, such as blood coagulation factor VIII for the extraction of DNA from Pseudorabies virus and production of capture antigen enzyme-linked immunosorbent assay (ELISA) when diagnosing Japanese Encephalitis (JE) [1,5,9].

In this research, the effect of different culture media composition and different serum levels was investigated by using the Taguchi experimental design method with four-level factors for suspension cultures. The effects of each supplement and its levels on growth and proliferation of BHK cells are essential. This study aimed to optimize the conditions for BHK cell culture in the desired conditions.

## Material and Methods

### Growth and Maintenance of BHK Cells

The cells used were Industrial BHK received from the Department of Foot-and-Mouth Disease in the Razi Vaccine and Serum Research Institute (RVSRI), Karaj, Iran. These cells were grown and propagated in modified minimum essential media (MEM) by adding a set of proteins including Lactalbumin (2.50g/l), yeast extract (1.00g/l), peptone (2.50g/l), New Zealand casein (1.00g/l); Glutamine (0.50g/l )and 2% sodium bicarbonate were also added to the culture medium. Also, 5-10% of the serum treated with polyethylene glycol 6000 was added to modified MEM. 100 IU of penicillin G and streptomycin for controlling microbial load was added as well. After keeping the BHK cells in a liquid nitrogen tank and passing them through the preparation steps, the cells were transferred to T-flasks for subsequent cultivation. The flasks were kept in incubators at an operating temperature of 36.5°C and 5% carbon dioxide. Forty-eight hours after culturing the cells, the flasks were removed from the incubator, and if the morphology and surface were appropriate, cells were removed from the flasks by using trypsin-versene 2.5% and transferred to the bioreactor. The used bioreactors possessed a volume of 1.0 liter and also had U-shaped blades and air exchange vents. Stirring was done at a speed of 110-130 rpm, and the starting pH of the culture ranged from 7.1 to 7.2 while carbon dioxide was used to control the pH. The initial cell seeding density was 3.5 * 10^+5^ per ml. The cells had undergone 3–5 trypsinizations in this condition before entering the experimental stage. All experiments were conducted in three replications. Cells sampling was done at intervals of 24, 48 and 72 hours after beginning cultivation to examine the cell’s position in terms of cell count and pH.

### Experimental Design

In order to evaluate the effect of different supplements, six types of various kinds (each having four different levels on growth and proliferation of BHK cells in the suspension culture), it would be necessary to conduct 10296 experiments, but with the use of the Taguchi method, this number is reduced to 32. Apart from reducing the number of tests and saving time and costs, this method can also determine the effect of the factors in each level and determine the optimal result. In this study, this method was used to verify the impact on the number of final cells of six factors using four levels (Table 1). Using the Taguchi design experimental method with the Design of Experiments software, the number of experiments based on six variables at four levels is described in Table 2.

**Table 1:** Number of factors and levels.

**Symbols**	**Modified MEM factors**	**Levels**			
**A**	Calf serum (%)	1.00	2.00	5.00	10.00
**B**	Glucose (gr/l)	1.00	2.00	4.00	6.00
**C**	Glutamine(gr/l)	0.25	0.50	0.75	1.00
**D**	Mixture of Protein(gr/l)	0.96	3.50	7.50	10.06
**E**	Mixture of Vitamin(gr/l)	1.50	3.00	6.00	12.00
**F**	Mixture of Essential Amino acids(gr/l)	1.50	3.00	6.00	12.00

**Table 2:** Design Experiment Chart by Taguchi Method.

**Exp.No**	**Levels**					
	**Calf serum (%)**	**Glucose (gr/l)**	**Glutamine (gr/l)**	**Protein (gr/l)**	**Vitamin (gr/l)**	**Essential Amino acid (gr/l)**
**1**	1.00	1.00	0.25	0.96	1.50	1.50
**2**	1.00	2.00	0.50	3.50	3.00	3.00
**3**	1.00	4.00	0.75	7.00	6.00	6.00
**4**	1.00	6.00	1.00	10.06	12.00	12.00
**5**	2.00	1.00	0.25	3.50	3.00	6.00
**6**	2.00	2.00	0.50	0.96	1.50	12.00
**7**	2.00	4.00	0.75	10.06	12.00	1.5.00
**8**	2.00	6.00	1.00	7.00	6.00	3.00
**9**	5.00	1.00	0.5.00	7.00	12.00	1.50
**10**	5.00	2.00	0.25	10.06	6.00	3.00
**11**	5.00	4.00	1.00	0.96	3.00	6.00
**12**	5.00	6.00	0.75	3.50	1.50	12.00
**13**	10.00	1.00	0.50	10.06	6.00	6.00
**14**	10.00	2.00	0.25	7.00	12.00	12.00
**15**	10.00	4.00	1.00	3.50	1.50	1.50
**16**	10.00	6.00	0.75	0.96	3.00	3.00
**17**	1.00	1.00	1.00	0.96	12.00	3.00
**18**	1.00	2.00	0.75	3.50	6.00	1.50
**19**	1.00	4.00	0.50	7.00	3.00	12.00
**20**	1.00	6.00	0.25	10.06	1.50	6.00
**21**	2.00	1.00	1.00	3.50	6.00	12.00
**22**	2.00	2.00	0.75	0.96	12.00	3.00
**23**	2.00	4.00	0.50	10.06	1.50	3.00
**24**	2.00	6.00	0.25	7.00	3.00	1.50
**25**	5.00	1.00	0.75	7.00	1.50	3.00
**26**	5.00	2.00	1.00	10.06	3.00	1.50
**27**	5.00	4.00	0.25	0.96	6.00	12.00
**28**	5.00	6.00	0.50	3.50	12.00	6.00
**29**	10.00	1.00	0.75	10.06	3.00	12.00
**30**	10.00	2.00	1.00	7.00	1.50	6.00
**31**	10.00	4.00	0.25	3.50	12.00	3.00
**32**	10.00	6.00	0.50	0.96	6.00	1.50

### Count, morphology and pH of cells

Cell counting was performed using a hemocytometer (improved Neubauer), and Trypan blue exclusion every 24h (1:1 mixture of 0.2% Trypan blue in normal saline solution and sample). Unstained cells were considered viable, while stained cells were deemed to be dead. Additionally, their condition was examined to determine the appearance of the cells after each sampling under the inverted microscope [10].

To determine the pH, a sample of the cells that trapped the entire volume of the tube (in order to prevent pH changes - for example, in case of incomplete mixing with the air), was examined using a pH tester (Hanna, Spain).

## Results

**Figure 1:**
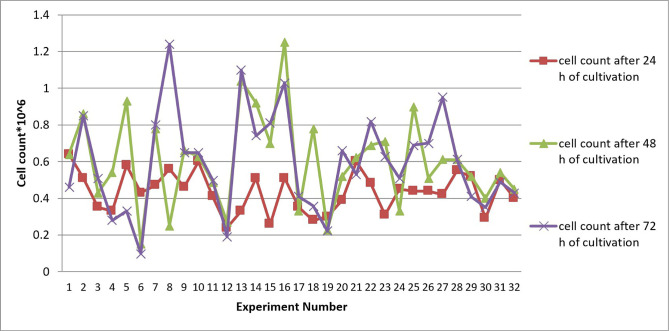
Cell count of 32 runs of experiment at 24, 48, 72 hours after cultivation.

The average number of cells is shown in Figure1 at 24, 48 and 72 hours after culturing the cells. The average pH of each of the three replications was also similar to the number of final cells. In order to carry out the experiment, cells were seeded at 3.5 ×10^+5^ using 1-liter bioreactors. The initial pH was 7.1 ± 0.1 and the cells were stirred at 125 ± 10 rpm (Figure 1).

### The effect of various supplements on the cell count and pH

**Figure 2:**
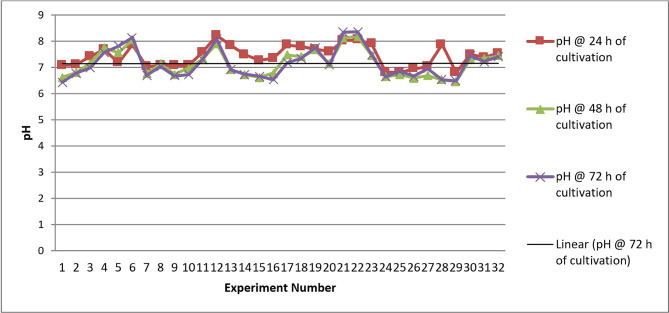
pH of 32 runs of experiment at 24, 48, 72 hours after cultivation.

During the study, industrial BHK cells were cultivated in 32 different media. The hypothesis was to investigate the effects of different media composition on cell count and pH (Figure 2).

### The effect of polyethylene glycol-treated calf serum

Four different levels were used to evaluate the effect of the serum on the growth and proliferation of BHK cells (Table 1). The percentages of tested serum were 1%, 2%, 5% and 10%.

As expected, by increasing serum concentration, cell culture status was improved in terms of number, apparent cellular quality, homogeneity of the cytoplasmic membrane. These results are in accordance with the ones obtained by other researchers [11].

The experiment was concluded by using Taguchi methods of experimental design, at three levels of cultivation. Also, in the other investigations, it was discovered that by increasing the serum level, the cells’ condition (which includes morphology) could also be observed [4, 12, 13].

 An essential point regarding suspension culture is the much larger cell population that was determined in the experiments. The highest number of cells after 72 hours was related to the eighth test, where cell count equaled 1.24 * 10^+6^ cells per ml, in which the amount of serum treated with polyethylene glycol was 2%. On the other hand, by increasing serum levels, the pH was more resistant to changes in culture media.

### The effect of proteins

The animal serum has numerous problems, which includes the high potential for contamination and increasing the cost of its extraction process. Proteins are a significant part of the animal serum; using synthetic proteins can reduce the use of serum. The effect of proteins on the growth and proliferation of cells was studied as well; proteins including lactalbumin, yeast extract, peptone and New Zealand casein, were used at four different levels (Table 1).

The amounts of protein consumed in the tested culture media were 0.96, 3.50, 7.00 and 10.06 g/l.

By increasing the total protein content, it was expected that the number of final cells should also increase. By decreasing the protein content, the pH of the culture medium was increased which is not appropriate – an increase in pH results in a loss of cell culture quality, including cell count and morphology. The pH of culture number 16 was equal to 6.78 in 48 hours after initiation of the culture and therefore suitable for BHK cell cultures.

## The effect of vitamins

The effects of vitamins, like all the other factors, were studied in four levels (Table 1). The amount of vitamin consumed in the culture medium was 1.50 g/l at the first level, at the second level it was 3.00 g/l, at the third it was 6.00 g/l and 12.00 g/l at the fourth.

Contrary to the general notion, the highest number of cells was observed at level 2. In fact, this indicates that by increasing vitamin levels, we do not necessarily have more cells. However, it can be stated that vitamins in the serum can also contribute to the growth and proliferation of cells. In test number 8 and 13, whose final cell count was similar, the amount of vitamin was 6.00 g/l. In test number 7, 11 and 15, the amount of vitamin was the lowest, the pH of the last test after 72 hours was proper, but the number of final cells was not suitable.

### The effect of essential amino acids

The number of essential amino acids used in the tested culture media was 1.50 g/l at the first level, 3.00 g/l at the second level, 6.00 g/l at the third and 12.00 g/l at the fourth level.

The essential amino acid mixture contains l-Arginine, l-Cysteine, l-Cystine, l-Histidine, l-Isoleucine, l-Leucine, l-Lysine, l-Methionine, l-Phenylalanine, l-Threonine, l-Tryptophan, l-Tyrosine and l-Valine [14]. Some essential amino acids, such as l-Tyrosine were proposed to be crucial for cell growth [15,16].

As the amount of essential amino acid mixture in the specified composition was increased, the number of final cells increased, and this was the first hypothesis of our research. During the experiments, it was determined that an increase in the number of essential amino acids did not increase the number of final cells, which could be due to the presence of serum and protein in the culture medium. In fact, it can be predicted that the presence of proteins will reduce amino acid liability in the medium. Certainly, the mentioned amino acids are all essential amino acids except glutamine. The pH value is related to the number of used amino acids. The relationship is hardly predictable due to the interference of other agents. Overall, by increasing the number of amino acids at 12.00 g/l, the pH was more than 7.50 g/l in most experiments, which is by no means suitable for growth and proliferation of cells.

### The effect of glucose

The amount of glucose consumed in the culture medium was 1.00, 2.00, 4.00 and 6.00 g/l. By decreasing the amount of glucose from 6.00 g/l to 1.00 g/l, contrary to the initial prediction, an increase in the number of cells and the pH stability was observed. Glucose is vital in the culture medium, and its absence can disrupt the process of growth and proliferation of cells. The optimum glucose level in this culturing process was 1.00 g/l, which is a significant difference in the amount of glucose consumed in the culture media in other studies on BHK cells [10]. By increasing glucose levels, there was no increase observed in BHK cells. However, lactate levels were increased, which is damaging to cell growth and proliferation. Most research concluded that the lack of glucose, or its reduction, leads to cell death, and BHK cells have a high rate of glucose consumption. Based on these results, it can be concluded that glucose influences the ph value considerably. The absence of glucose increases the culture medium pH and, as a result, induces cell death. In this study, the amount of used glucose varied in tests 8, 13 and 16, but in the medium which had the highest number of cells before 72 hours have passed and proper pH value, the glucose level was 1.00 g/l.

## The effect of Glutamine

Due to the importance of this molecule, the effect of glutamine alone is usually studied. The amount of glutamine in the culture media used during the experiments ranged from 0.25 g/l to 1.00 g/l. Other glutamine levels were also used at intervals of 0.25 g/l. In this study, it was discovered that increasing the amount of glutamine in the culture medium did not increase the number of final cells. The results of other researchers indicate that by increasing the amount of glutamine in the medium, the rate of consumption but not necessarily productivity increased [10]. By increasing the amount of glutamine in the culture medium, the amount of ammonium was increased. Ammonium decreases cell growth rate and proliferation, which can have a negative effect on crop production in the cell culture process. Generally speaking, a medium with 0.25 g/l glutamine does not have a high number of cells after 48 or 72 hours after culturing the cells. The pH value is appropriate for the growth and proliferation of cells with the amount of consumed glutamine. In order to achieve a proper pH, it is necessary to determine the amount of glutamine used according to the culture conditions. On this basis, the most suitable pH for cell culture in most experiments is achieved when the glutamine amount equals 0.75 g/l.

### Comparing the optimum culture medium by using the Taguchi method

**Figure 3:**
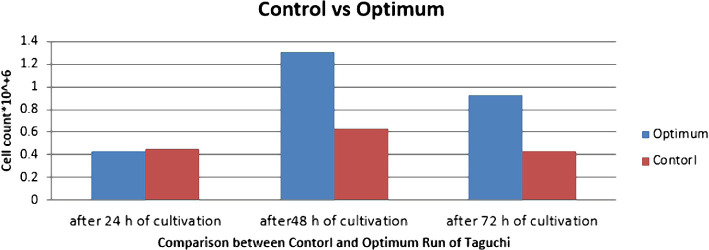
Comparison of cell count between optimized and control media.

**Figure 4:**
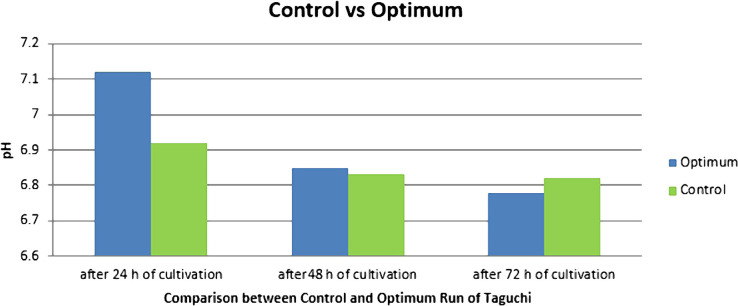
Comparison of pH values between optimized and control media.

According to Table 3, which indicates the number of cells at different hours and the pH value in the culture medium when using the Taguchi design method and the usual culture media, it is observed that the number of cells in the optimized media was more than double at 48 hours after culturing the cells while the pH value was similar in the two media. Also, the protein content of essential amino acids can be reduced by 50%. Reduction in the use of glucose, essential amino acids and proteins do not have any influence on the number of final cells, which indicates the success rate in suspension cell cultures. When reducing these three supplements, there was no significant change in the pH value compared to the usual culture medium, and the difference was only 0.04 at 72 hours after culturing the cells (Table 3), (Figure 3 and 4).

**Table 3:** Comparison between media composition of optimized and control media.

**Media**	**Calf serum (%)**	**Glucose (gr/l)**	**Glutamine (gr/l)**	**Protein Mix (gr/l)**	**Vitamin Mix (gr/l)**	**Essential Amino Acid Mix (gr/l)**	**Cell count (cells/ml) (×10**+6**)**			**pH**		
							**24hr**	**48hr**	**72hr**	**24hr**	**48hr**	**72hr**
**Optimization**	10.00	1.00	0.75	3.50	6.00	3.00	0.43	1.31	0.93	7.12	6.85	6.78
**Control**	2.00	4.00	0.50	7.00	6.00	6.00	0.45	0.63	0.43	6.92	6.83	6.82

## Discussion

The success rate of culturing suspension cells is always investigated based on the measurement of the final cells number. Therefore, at each stage of the culturing process, it can be stated that any experiment with a higher number of final cells is a more suitable culture. The effects of chemical variables including serum, glucose, essential amino acids, glutamine and vitamins, all of which supplemented minimum essential media, as well as protein content including lactalbumin, yeast extract, New Zealand casein and peptone, were studied in order to determine the optimum culturing conditions.

At 24 hours after culturing the cells, the highest number of final cells was obtained for tests number 1 and 10. The only common point between these two cultures was that they had an equal glucose level of 0.25 g/l. In experiments number 8 and 16, the highest number of cells was observed at 48 hours after culturing and was equal to 1.25 * 10^+6^ Glucose and essential amino acids were in the same amount in the two media. In the majority of cultures, the number of final cells at 48 hours increased relative to the number of final cells at 24 hours after culturing the cells, and test number 8 had the highest number of cells at 72 hours (1.25 * 10^+6^. As observed in the results table, most of the experiments recorded a greater demographic decline at 72 hours compared to 24 hours after culturing the cells.

In a study by Blaker et al. on the requirements of glucose, glutamine and insulin of HeLa cells for growth in suspension culture, it was found that the effect of serum on growth and proliferation of cells was positive and improved the culture success rate [17]. In this study, the same amount of glucose with an increase in the amount of amino acid has increased the cell population by up to 50% but has not affected the doubling time. Glutamine is an essential nutrient for cell growth in vitro condition. Adding glutamine to the culture medium and increasing the amount of glutamine increases the amount of ammonium. Based on the research conducted by Hecneman et al., it has been determined that reducing the number of amino acids can affect the growth and proliferation of cells [19]. Particularly, certain amino acids such as glutamine, which have high cell consumption rates, are more effective than other amino acids. The primary energy source for the activity of cells is glucose and glutamine. According to Neerman et al., the consumption rate of glucose and glutamine in mammalian cells are similar to each other [19]. The Taguchi design technique used in this research was found to be efficient in the development of an optimized medium for baby hamster kidney cells in suspended cultures in order to increase the cell population. Medium supplements which had positive effects on cell growth and proliferation were identified in only 32 tests, and these supplements were glutamine, serum, vitamin, essential amino acids, proteins, and glucose. Based on the parameters identified using the Taguchi method, an optimized medium for BHK cells in suspended cultures has been formulated. The factors that were considered in this article in order to achieve an optimized media were comparable to the findings of previous researchers [20]. Based on a study conducted by Cot’e et al., it can be noted that different levels of glucose and glutamine can be effective in the production of metabolites such as ammonium and lactate that have inadequate effects on cells growth and proliferation so that by reducing the amount of glucose and glutamine, a large amount of harmful metabolites can be omitted. Therefore, the amount of glutamine and glucose should be reduced and optimized [3]. Heidemann et al. found that the addition of peptone, which is one of the commonly used proteins in the culture medium, can improve cell production [21]. Peptone is one of the protein complexes in our research and it has a positive effect on the growth and proliferation of cells, according to our research. However, in our study, we have also been found the optimum levels of protein mixture for culturing BHK cells.

## Acknowledgement

The authors are profoundly grateful to the Foot and Mouth Disease Reference Laboratory, Razi Vaccine & Serum Research Institute, Agricultural Research Education and Extension Organization (AREEO), Karaj, Iran for imparting knowledge, assistance and training regarding the advanced techniques.

## Conﬂict of Interest

The authors confirm that there are no conﬂicts of interest.
